# Psychological Flourishing Experiences Among Burn Patients: A Phenomenological Qualitative Study Informed by the PERMA Framework

**DOI:** 10.3390/healthcare14213633

**Published:** 2026-10-27

**Authors:** Donglan Zhang, Wanling He, Shuyao Zhang, Yuan Wang, Hui Yu, Liying Chen, Mengmiao Peng, Haiyan Li

**Affiliations:** 1 School of Nursing, Guangdong Medical University, Dongguan 523808, China; zhangdonglan23@163.com; 2 Department of Burns, Guangzhou Red Cross Hospital of Jinan University, Guangzhou 510220, China; 3 Department of Pharmacy, Guangzhou Red Cross Hospital of Jinan University, Guangzhou 510220, China

**Keywords:** flourishing, burn, PERMA model, experiences, qualitative

## Abstract

**Highlights:**

**What are the main findings?**

- Burn patients’ flourishing involved positive emotions, active engagement, multilevel support, meaning reconstruction, and renewed accomplishment.
- Flourishing was shaped by the interplay of psychological adjustment, rehabilitation experiences, and social support.

**What are the implications of the main findings?**

- Burn care should incorporate positive psychological well-being alongside physical recovery and symptom management.
- Integrated family, healthcare, and social support may facilitate psychological adaptation and active engagement in rehabilitation.

**Abstract:**

**Background/Objectives**: Burns can impair physical functioning and exert long-term effects on mental health and quality of life. The PERMA framework conceptualizes psychological flourishing across positive emotion, engagement, relationships, meaning, and accomplishment, providing a useful lens for understanding positive psychological experiences during burn recovery. This study aimed to explore psychological flourishing experiences and the factors shaping these experiences among patients with burns from the perspective of the PERMA framework and to inform positive psychological support strategies. **Methods**: A phenomenological qualitative design was employed. Participants were recruited using purposive sampling. Twenty-two hospitalized patients with burns were recruited from the burn department of a tertiary hospital in Guangzhou, China, between December 2025 and March 2026. Semi-structured interviews were conducted using an interview guide informed by the PERMA framework. Interviews were transcribed verbatim and analyzed using Colaizzi’s seven-step method. **Results**: Five themes and 14 subthemes were identified: (1) reconstruction of positive emotions, including restoration of hope, development of positive cognition, and acceptance of reality; (2) active engagement, including participation in rehabilitation, reconstruction of daily functioning, and social participation; (3) multilevel support systems, including family, healthcare team, other-person, and social security support; (4) reconstruction of meaning in life, including transformation of life attitudes and family responsibility-driven motivation; and (5) new experiences of accomplishment, including a sense of achievement through progress and expectations for future life. **Conclusions**: Psychological flourishing among patients with burns involves multidimensional psychological and social experiences that can be interpreted through the PERMA framework. Nursing care may benefit from greater attention to patients’ positive psychological resources, as well as greater consideration of family, healthcare, and social support when addressing psychological adaptation and engagement in rehabilitation.

## 1. Introduction

Burn injury is a common and severe trauma that can damage skin and deep tissues, impair function, and result in long-term disability, substantially reducing patients’ quality of life [1]. According to the World Health Organization (WHO), approximately 180,000 deaths worldwide are attributable to burns each year. With continuous advances in burn treatment and nursing care, survival rates among burn patients have steadily improved, while overall hospitalization duration and mortality rates have declined [2]. However, these improvements in survival outcomes have not been accompanied by corresponding gains in psychological well-being [3]. Growing evidence indicates that burn patients continue to experience persistent and complex psychological distress throughout rehabilitation. The prevalence of depression among burn patients has been reported to reach 60.7% [4], while the prevalence of post-traumatic stress disorder ranges from 9% to 45.2% [5]. In addition, burn survivors have been reported to exhibit elevated suicidal ideation and a higher lifetime prevalence of suicide attempts than the general population [6], and psychological recovery often lags behind physical rehabilitation [7]. These studies have primarily focused on negative psychological outcomes, such as anxiety and depression. Although such studies have contributed to the identification of psychological risk factors, they have, to some extent, overlooked the positive psychological experiences and internal strengths that individuals may develop following major trauma. As early as the 1950s, scholars cautioned that mental health models relying solely on negative indicators may hinder a comprehensive understanding of psychological functioning [8]. Accordingly, traditional assessment frameworks have emphasized the inclusion of positive indicators to more fully reflect individuals’ psychological well-being [9]. Therefore, it is necessary to explore the positive psychological states experienced by burn patients following trauma, as well as the factors influencing these experiences, from a more holistic perspective.

With the development of positive psychology, mental health research has gradually shifted from a disease-oriented paradigm focused on illness and deficits to a health-oriented paradigm. This paradigm shift highlights psychological adaptation and growth in the face of adversity [10,11]. Within this theoretical context, positive psychological indicators such as well-being, life satisfaction, and flourishing have become integral components of the concept of mental health and have been shown to be associated with long-term rehabilitation outcomes and social functioning recovery [12]. In burn care settings, increasing attention has been paid not only to psychological distress such as anxiety and depression, but also to patients’ positive psychological changes following trauma [13]. A qualitative study by Dukes et al. (2022) demonstrated that burn patients may exhibit multiple forms of positive adaptation during rehabilitation, including proactive coping, meaning-making, altruistic behaviors, self-acceptance, and active seeking of social support, all of which contribute to improved quality of life [14]. Similarly, Wu et al. (2026) explored the illness coping experiences of patients with moderate-to-severe burns, highlighting both the challenges patients faced and the critical roles of positive psychological qualities and family and social support in the coping process [15]. A cross-sectional study further revealed that, despite substantial physical and psychological challenges, some burn patients exhibit relatively high levels of psychological resilience [16]. In addition, life satisfaction has been recognized as an important outcome indicator in burn rehabilitation [17]. However, previous studies have primarily focused on positive adaptation or illness coping processes, with limited attention to flourishing as a multidimensional positive psychological state among patients with burns. Further exploration of their positive psychological experiences is therefore warranted.

In this context, standardized quantitative instruments, such as the PERMA-Profiler, have been developed to assess multidimensional flourishing and applied in clinical populations to examine levels of flourishing and their associations with psychosocial and health-related factors [18,19]. However, such measures may provide incomplete insight into how patients with burns subjectively experience and interpret flourishing within the specific contexts of injury, treatment, and rehabilitation. Flourishing is a multidimensional positive psychological construct encompassing positive emotion, engagement, relationships, meaning, and accomplishment [20]. A phenomenological qualitative approach is therefore well suited to exploring how these dimensions are perceived and expressed within the lived experiences of patients with burns [21]. A growing body of evidence suggests that higher levels of flourishing are not only closely associated with better mental health status and life satisfaction [22], but also serve as a buffer against negative psychological outcomes such as depression and suicidal ideation [23,24]. Furthermore, as a key positive psychological resource, flourishing contributes to improved adaptability and psychological resilience [25]. Accordingly, informed by the PERMA framework, the study aims to explore psychological flourishing experiences among burn patients during treatment and rehabilitation, and to provide insights into the development of positive psychology-informed nursing interventions.

Seligman (2019) proposed a shift in psychological research from a focus on illness and dysfunction toward human strengths and well-being [20]. Subsequently, he developed the PERMA model in 2011, which conceptualizes flourishing across five dimensions: positive emotions, engagement, relationships, meaning, and accomplishment [20]. Positive emotion refers to the experience of positive feelings such as hope, optimism, and joy; engagement reflects active participation in and concentration on meaningful activities; relationships denote supportive connections with significant others, including family members, friends, and healthcare professionals; meaning refers to a sense of purpose, value, and direction in life; and accomplishment reflects the satisfaction derived from achieving goals, making progress, or overcoming difficulties. Together, these five dimensions constitute core components of positive psychological functioning and overall well-being. The model emphasizes the synergistic effects of multidimensional positive psychological components, providing an integrated theoretical framework for understanding mental health [26]. Existing research suggests that each PERMA dimension contributes independently to individual well-being and is both measurable and amenable to intervention [27]. This provides a foundation for assessing and enhancing flourishing. Moreover, the PERMA model has been widely applied in nursing and health-related research across diverse cultural contexts. It has demonstrated good stability and applicability [19,28,29]. Intervention studies based on this model show that it can enhance positive emotions and subjective well-being. It can also alleviate psychological distress and negative emotions to some extent [30,31]. The PERMA framework therefore provides a multidimensional lens for systematically understanding psychological flourishing.

Although positive psychology has been increasingly applied in the context of chronic diseases, research examining psychological flourishing among patients with burns through the lens of the PERMA framework remains limited. Flourishing refers to a state of well-being that extends beyond the absence of illness [32]. Psychological constructs, including anxiety, depression, and flourishing are commonly assessed using standardized scales [18,22]. Although these instruments can efficiently generate standardized scores, they have limitations in capturing the subjective experiences and specific manifestations of flourishing among patients with burns in the context of sudden traumatic injury. Therefore, a patient-centered phenomenological qualitative approach informed by the PERMA framework may provide a more in-depth understanding of flourishing in this population. Previous studies based on the PERMA model have demonstrated that flourishing experiences are highly context-specific across different populations. For example, Song et al. (2025) reported that flourishing in lung transplant recipients was characterized by mindset shifts, meaning in life, health benefits, perceived support, and unmet needs [33]. Thurman et al. (2019) highlighted that flourishing among rural adults with disabilities involved multiple dimensions, including values, life attitudes, needs, and social participation [34]. Similarly, Oddiri et al. (2023) demonstrated that positive emotions, relationships, and meaning played important roles in promoting flourishing among healthcare workers [35]. Collectively, these findings suggest that flourishing experiences vary by population, disease type, and life circumstances. Notably, burns represent a sudden traumatic event in which patients face both acute stress reactions and long-term psychological adaptation challenges [36]. Accordingly, their positive psychological experiences may differ from those of other patient populations. Exploring psychological flourishing among patients with burns may help deepen understanding of psychological adaptation during treatment and recovery.

This study aimed to explore the psychological flourishing experiences of patients with burns, informed by the PERMA framework. Specifically, the study sought to examine the nature and multidimensional characteristics of these experiences and to explore the perceived factors shaping them during treatment and rehabilitation.

## 2. Materials and Methods

### 2.1. Design

A phenomenological qualitative study design was employed [37]. This approach is well-suited for revealing the essence of phenomena as experienced by research subjects. It focuses on individuals’ subjective experiences and how they construct meaning within their specific contexts. This approach facilitates a deeper understanding of psychological flourishing experiences among patients with burns. Furthermore, the Consolidated Criteria for Reporting Qualitative Studies (COREQ) checklist was followed to report this study [38]. Details can be found in Supplementary Material File S1.

### 2.2. Participants and Setting

Data collection took place between December 2025 and March 2026. Purposive sampling was used to recruit hospitalized burn patients from the Burn Department of a tertiary hospital in Guangzhou, China. The principle of maximum variation was followed during the sampling process, with consideration given to differences in gender, age, and educational level to ensure sample representativeness. The inclusion criteria were as follows: (1) patients with second-degree burns or above [39]; (2) at least 48 h post-burn injury and beyond the acute burn shock/resuscitation phase [40]; (3) aged 18 years or older; (4) conscious and able to understand effectively during the interview; and (5) willing to participate and provide written informed consent. Exclusion criteria included: (1) concurrent organic diseases, such as severe cardiac, hepatic, or renal insufficiency; and (2) cognitive impairment, communication difficulties, or other reasons that would prevent completion of the interview. The sample size was determined dynamically in accordance with qualitative research principles, with data saturation being continuously assessed as data collection and analysis proceeded in parallel. Data saturation was considered achieved when consecutive interviews yielded no new themes or information [41].

A total of 24 patients with burns who met the inclusion criteria agreed to participate in the study. Two participants withdrew during the interview process: one did not wish to continue the in-depth discussion, and the other experienced marked emotional distress when discussing personal experiences. In the latter case, the interview was terminated in accordance with the participant’s wishes to protect their emotional well-being. Finally, 22 participants completed the study and were included in data analysis; of these participants, 13 were men and 9 were women. All participants underwent one-to-one semi-structured interviews conducted in Chinese. Data collection and analysis proceeded concurrently. After the interview with the 20th participant, no new themes or substantively new information emerged. An additional interview was therefore conducted with the 22nd participant to confirm data saturation; no new themes or important information was identified, indicating that saturation had been achieved. Furthermore, one participant shared relatively limited information in the initial interview, so a second interview was carried out with this participant to collect more comprehensive data.

### 2.3. Research Team

The research team had multidisciplinary clinical and research backgrounds. D.Z. and W.H. had received qualitative research training, had prior interviewing experience, and had completed more than one year of clinical practice in the burn unit; they were primarily responsible for the interviews and qualitative analysis. Y.W., H.Y., and L.C. were senior nursing professionals with extensive experience in burn care, while M.P. had substantial surgical clinical experience and assisted with the interviews. S.Z. and H.L. provided methodological and research supervision, and burn surgeons supplied relevant clinical information when necessary to support interpretation of participants’ clinical conditions.

### 2.4. Data Collection

Data were collected using a face-to-face, one-on-one semi-structured interview method. The preliminary interview guide was developed with reference to the PERMA framework. Its five domains—positive emotion, engagement, relationships, meaning, and accomplishment—served as broad areas of inquiry to guide the development of open-ended interview questions. This approach also allowed participants to describe experiences beyond these domains. We also drew on a review of relevant literature and discussions within the research team. The interview content focused on burn patients’ positive psychological experiences during rehabilitation. Before the formal interviews, we conducted a pilot test with two burn patients. The pilot test assessed the comprehensibility and applicability of the interview guide. Based on participant feedback, we revised the wording of certain questions. Data from the pilot interviews were not included in the final analysis. The final semi-structured interview guide consisted of five core questions, which were presented in Table 1.

The interviews were conducted by D.Z. and W.H. Before the interviews, the researchers reviewed the participants’ demographic and clinical information and contacted them to arrange the interviews. Suitable interview times and locations were then agreed upon with the participants. The interviews took place in a relatively quiet and private area within the burn ward, creating a relaxed and safe environment for communication. After obtaining participants’ informed consent, all interviews were audio-recorded, and key non-verbal information was simultaneously documented. During the interviews, participants were encouraged to share their experiences and feelings freely. The researchers used appropriate probes based on the interview flow to elicit richer and more in-depth data. Interviews generally lasted approximately 30–50 min, with the duration adjusted according to each participant’s physical and emotional tolerance. After each interview, the researchers assisted participants in completing a general information questionnaire. All audio recordings were transcribed verbatim within 24 h following the interviews to ensure timeliness and accuracy of the data.

### 2.5. Data Analysis

Data analysis was conducted using Colaizzi’s seven-step phenomenological method [42]. During data analysis, the PERMA framework served as a sensitizing theoretical framework rather than as a fixed coding scheme. The specific meaning units, initial codes, and subthemes were generated from participants’ interview narratives following the phenomenological analytic process. After the subthemes had been developed, the researchers examined their conceptual correspondence with the PERMA framework and subsequently organized them into higher-order thematic structures.

The coding and theme extraction were performed independently by two researchers (D.Z. and W.H.), followed by discussion to reach consensus. The specific analytical process was as follows: (1) the researchers repeatedly read the verbatim transcripts to gain an overall understanding of the participants’ experiences; (2) statements directly related to participants’ psychological flourishing experiences were identified and extracted; (3) the researchers interpreted significant statements and formulated initial meaning units while remaining grounded in the original narratives; (4) related meaning units were compared, grouped, and synthesized into subthemes and broader thematic categories; (5) the identified themes and subthemes were integrated into a comprehensive description; (6) the description was further condensed into a coherent thematic structure; and (7) the preliminary thematic structure was returned to selected participants for member checking, with feedback considered in refining the themes where appropriate. Throughout the analytical process, the research team iteratively revised the thematic structure through ongoing comparison and discussion, ensuring that the themes accurately reflected the authentic experiences of the participants. When disagreements arose between the two researchers, a third researcher was involved in the discussion to reach a consensus.

### 2.6. Rigour and Reflexivity

To enhance the rigour and trustworthiness of the study, multiple quality assurance strategies were implemented throughout data collection and analysis. All interview recordings were transcribed verbatim within 24 h after each interview and repeatedly cross-checked against the original audio recordings to ensure transcription accuracy and completeness. Data collection and analysis were conducted concurrently, allowing emerging insights to inform subsequent interviews and facilitate deeper exploration of participants’ experiences.

To improve the credibility of the findings, after the preliminary themes had been developed, the findings were returned to selected participants for member checking to determine whether the interpretations accurately reflected their experiences. Their feedback was considered in refining the themes where appropriate. In addition, representative quotations were retained during data analysis to ensure that the themes remained grounded in participants’ original narratives and to preserve the authenticity of their experiences.

To enhance the dependability and confirmability of the analysis, two researchers independently conducted coding and theme development. Discrepancies in interpretation were discussed within the research team until consensus was achieved, thereby strengthening the consistency and stability of the analytical process. Furthermore, an audit trail was maintained throughout the study to systematically document methodological decisions, coding procedures, and theme refinement processes, ensuring transparency and traceability of the research process.

Reflexivity was maintained throughout the study. Following each interview, researchers documented reflexive notes to critically examine their assumptions, prior experiences, and potential subjective biases related to burn rehabilitation and psychological recovery. Through regular team discussions and reflective dialogue, researchers continually reassessed how their perspectives might shape data interpretation, thereby helping to reduce potential interpretive bias and enhance the trustworthiness of the findings.

### 2.7. Ethical Considerations

Ethical approval for this study was obtained from the relevant institutional ethics committee (Approval No. 2025-402-01). The study adhered strictly to ethical principles including informed consent, confidentiality, and respect for participant autonomy. Written informed consent was obtained directly from all participants after they had been fully informed of the study purpose, content, and potential risks. As all participants were conscious adults with sufficient decision-making capacity, consent from relatives was not required. During the data collection and processing phases, all interview data were managed using anonymous coding to ensure the confidentiality of participant identity information. Interview recordings and verbatim transcripts were used solely for the purposes of this study. They were securely stored by the research team with restricted access. After study completion, they were disposed of safely in accordance with relevant regulations.

## 3. Results

### 3.1. Characteristics of Participants

A total of 22 burn patients were included in this study and sequentially assigned identification numbers from P1 to P22. Among the participants, 13 were male (59.1%) and 9 were female (40.9%). The participants ranged in age from 19 to 80 years, with a mean age of 43.41 ± 16.97 years. No suicide attempts or deaths by suicide occurred among the participants during the study period. The general and disease-related data of burn patients are shown in Table 2.

### 3.2. Themes

**Figure 1:**
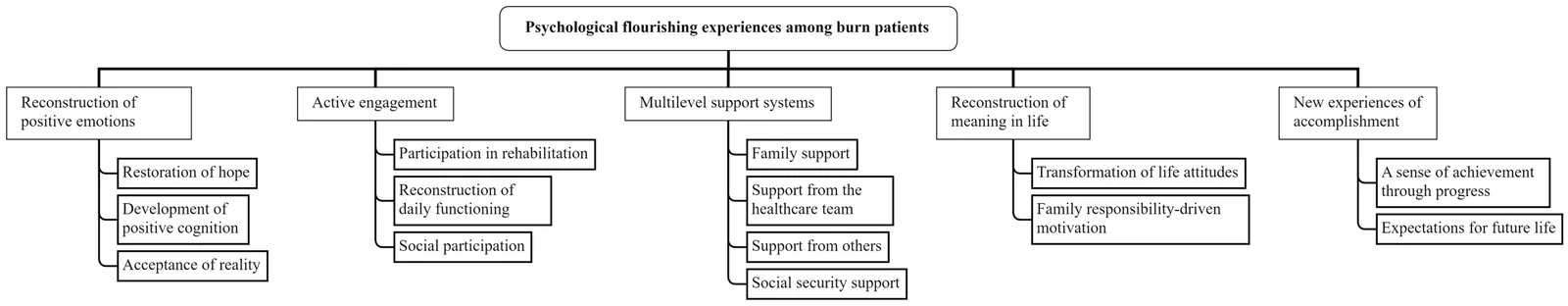
Themes and Subthemes Identified from the Interviews.

Through phenomenological analysis of the interview data, five core themes and their corresponding subthemes were identified. These themes were subsequently interpreted in relation to the PERMA framework. The five themes included: (1) reconstruction of positive emotions (restoration of hope, development of positive cognition, and acceptance of reality); (2) active engagement (participation in rehabilitation, reconstruction of daily functioning, and social participation); (3) multilevel support systems (family support, support from the healthcare team, support from others, and social security support); (4) reconstruction of meaning in life (transformation of life attitudes and family responsibility-driven motivation); and (5) new experiences of accomplishment (a sense of achievement through progress and expectations for future life). The core themes and corresponding subthemes derived from the data analysis are presented in Figure 1.

### 3.3. Theme 1: Reconstruction of Positive Emotions

Participants described negative emotions such as anxiety, fear, helplessness, and low mood in the context of sudden traumatic injury and prolonged treatment. At the same time, many participants also reported positive psychological experiences, including hope, optimism, and emotional stability, particularly in relation to perceived improvements in their condition and rehabilitation.

#### 3.3.1. Restoration of Hope

Most participants described perceived wound healing, positive feedback from healthcare professionals, and improvements in physical functioning as important sources of confidence in recovery. These experiences were associated with greater hope and more positive expectations regarding future recovery. Some participants also linked perceived improvements in their condition with reduced anxiety and uncertainty.

- **P13:** “At first, seeing how severe the wound was, I was terrified and worried about whether I’d recover. Later, as it healed bit by bit and the doctors said the recovery speed was good during rounds, the wound area kept shrinking. This filled me with hope.”
- **P14:** “The doctor explains the recovery status during every dressing change. They said if it continues like this, I might avoid surgery. I’m terrified of surgery, so hearing that made me very happy. I want to know more about my wound; it makes me feel grounded.”
- **P15:** “After the surgery, I could slowly move my left hand. It hurt at first, but now I can make a fist and extend my hand, and the pain is much less. I can feel the progress myself, which makes me very happy.”

#### 3.3.2. Development of Positive Cognition

Some participants described using self-encouragement, cognitive reframing, and reduced worry to maintain emotional stability during recovery. Focusing on the present rather than on uncertain future outcomes was commonly reported. Some participants also described not wanting to burden their families, which was linked to efforts to remain optimistic and resilient.

- **P9:** “I never expected to be burned or hospitalized, but I try to keep an open mind and think that tomorrow will be better. I can still move my fingers, so it’s not the worst-case scenario. I’m actually quite lucky.”
- **P17:** “The hardest days are behind me. I’m learning to adjust my mindset and not think too far ahead. I find things to do to distract myself and recover slowly. I tell myself, ‘at least I’m still alive.”
- **P1:** “My two daughters work, and I don’t want to trouble them. A good mindset leads to faster recovery. I just follow the doctors’ and nurses’ arrangements so I can be discharged sooner.”

#### 3.3.3. Acceptance of Reality

Some participants described being able to confront the physical changes and life impacts caused by the burn despite the distress associated with the injury. They also reported varying degrees of acceptance of their current physical condition. Although the burn event itself could not be changed, participants’ accounts reflected greater emotional stability and a sense of inner calm.

- **P6:** “I’ve been in treatment for over two months and have had more surgeries than I can count. I know I won’t return to how I was before; I’m mentally prepared for that. It’s not as painful as when I was first injured. I just want to recover soon.”
- **P21:** “My attitude now is—since it happened and I can’t change it, I can choose how to face it. I cooperate with the treatment every day and slowly accept this reality. Being anxious doesn’t help. By taking it step-by-step, my emotions have gradually calmed down.”

However, not all participants were able to readily accept the reality of their condition. Severe functional impairment or permanent physical changes were often accompanied by intense negative emotions and were perceived as adversely affecting psychological well-being.

- **P5:** “ At that time, I felt life wasn’t worth living. I didn’t dare look at my feet. The doctor said they’d try to save them, but my biggest fear is amputation. I can’t accept it; I cry every time I think about it.”

### 3.4. Theme 2: Active Engagement

Many participants described active involvement in rehabilitation rather than a purely passive role in care. This involvement included participation in functional training, resumption of activities of daily living (ADLs), and adaptation to physical changes, which were accompanied by a greater sense of self-care agency. Some participants also described engaging in social interactions or hobbies, which they associated with a greater sense of order in their daily lives.

#### 3.4.1. Participation in Rehabilitation

During the rehabilitation phase, many participants exhibited a proactive attitude toward treatment. They expressed a willingness to comply with clinical protocols and persist in functional exercises to accelerate recovery. This persistence not only facilitated physical restoration but also provided a sense of agency and achievement.

- **P13:** “I just need to cooperate with the doctors and nurses—the IVs, the medication, the rehab. I’m hoping my hand function recovers quickly.”
- **P10:** “I remember being too afraid to move in the beginning. Now I follow the therapist’s exercises every day—clenching, releasing, abducting. You have to control the force. It was hard at first, but by sticking with it, I feel more flexible. Persistence is key.”

#### 3.4.2. Reconstruction of Daily Functioning

Participants described engaging in basic ADLs, such as walking, eating, and performing simple chores, despite changes in physical functioning. These experiences were associated with a greater sense of independence and structure in daily life, as participants adapted their activities to their current physical condition.

- **P3:** “In the BICU (Burn Intensive Care Unit), I was bedridden and covered in dressings. Everything—eating, using the toilet—happened in bed. Now I can walk, eat, and bathe by myself. I’m satisfied and glad I don’t have to trouble others anymore.”
- **P22:** “My hand was severely burned; it’s deformed and won’t straighten completely. Eating is a bit inconvenient, but I can manage most other things. I’ve grown used to it and accepted the change. Life must go on.”

#### 3.4.3. Social Participation

Some patients expressed a growing desire to re-engage in social life, including returning to work, fulfilling family roles, or resuming social gatherings. Reintegration into society symbolized not only functional recovery but also a return to normalcy.

- **P19:** “My colleagues are waiting for me to go back. I want to return to work as soon as possible so my injury doesn’t affect the company’s affairs.”
- **P7:** “I love traveling. I had a trip planned with friends before the injury. Once I recover, I’ll follow through with those plans.”

### 3.5. Theme 3: Multilevel Support Systems

Participants perceived multi-level support from family, medical teams, peers, and social systems. These resources collectively mitigated psychological pressure and enhanced confidence in recovery.

#### 3.5.1. Family Support

Family was identified as the most vital and stable source of support. Family members provided both instrumental care (daily chores, bedside care) and emotional support (encouragement and comfort), profoundly influencing the recovery process.

- **P3:** “My son, who is in the military, took leave to see me. He stayed for three days and helped me decide on the surgery. Usually, we only video call, so having him here made me want to get better even faster.”
- **P12:** “My husband is at the hospital taking care of me almost constantly. He helps with everything since I can’t get out of bed. When I’m bored, he chats with me about the past. My parents are also very worried and visit despite their age.”

#### 3.5.2. Support from the Healthcare Team

Healthcare professionals played a crucial role by providing professional guidance and clear communication, which helped patients understand their progress and cooperate actively.

- **P19:** “I trust the doctors and nurses here. The doctor explains every step—when the dressing will be changed, when the surgery is, and where the skin graft will be taken from. It’s very reassuring.”
- **P4:** “The nurses are very attentive. Yesterday I had a fever, and they caught it immediately. The rehab therapist also showed me how to do hand exercises to avoid scar contracture. I’m truly grateful.”

#### 3.5.3. Support from Others

Beyond the immediate family, visits from friends and colleagues made patients feel valued. Additionally, peer support from other patients provided a unique sense of validation and shared experience.

- **P12:** “My aunt and other relatives traveled from far away to see me. Friends message me constantly. They even raised some money for my expenses; I was deeply moved.”
- **P7:** “I enjoy chatting with other patients and sharing my experience with scar prevention. Helping them makes me feel good about myself.”
- **P18:** “A few patients with similar injuries told me how they recovered step-by-step. Hearing them makes me feel that my situation is normal, which puts my mind at ease.”

#### 3.5.4. Social Security Support

Systemic support, such as health insurance and work-related injury compensation, alleviated the financial burden on families, allowing patients to focus on rehabilitation.

- **P11:** “I’ve been retired for a long time, and insurance covers most of my bills. Even though some items are out-of-pocket, the covered portion is significant, which makes me feel secure.”
- **P8:** “I was injured at work, so I have worker’s compensation. I don’t have to worry about money, and my boss said my job will be waiting for me.”

### 3.6. Theme 4: Reconstruction of Meaning in Life

Major trauma often prompts a re-evaluation of life’s value. Patients reflected on the fragility of life, becoming more appreciative of health and family responsibilities.

#### 3.6.1. Transformation of Life Attitudes

Reflecting on the trauma led many to realize the importance of health and the vulnerability of the human body. This resulted in a more cherished outlook on life and a focus on the present.

- **P5:** “I was just soaking my feet in hot water and didn’t think it was that hot. I ignored the redness until it turned black. I should have come to the hospital sooner. I’ll never be so careless again.”
- **P21:** “This experience made me rethink things. I won’t work myself to death anymore; safety and health are what truly matter.”

#### 3.6.2. Family Responsibility-Driven Motivation

Family duties served as a powerful driver for persistence in treatment. The need to raise children or support elderly parents reinforced the patients’ sense of self-worth and purpose.

- **P2:** “I have two kids to raise and my wife isn’t working. I’m the sole breadwinner. Their school fees depend on me, so I have to get treated.”
- **P20:** “My family even sold their house for my treatment. They tell me to think of my child and not give up. I know I must persist; I can’t let them down.”

### 3.7. Theme5: New Experiences of Accomplishment

Participants described functional improvements and the resumption of daily activities as meaningful accomplishments. These experiences were associated with greater confidence and clearer expectations and goals for future life.

#### 3.7.1. A Sense of Achievement Through Progress

The transition from dependency to independence provided visible evidence of progress, fostering a sense of pride and competence.

- **P18:** “In the beginning, I couldn’t even lift my hand. Now I can eat, dress, and go to the bathroom. This is huge progress for me and gives me the confidence to keep training.”
- **P16:** “Seeing these changes—from needing help with everything to walking on my own—actually gives me a great sense of achievement.”

#### 3.7.2. Expectations for Future Life

Participants established clear goals, such as returning to work, which symbolized a return to their pre-injury identity.

- **P1:** “My biggest goal now is to recover so I can go back to my old life—going to the park and having tea with my friends.”
- **P8:** “I’ve set a goal for my hand rehab. Once I’m better, I plan to go back to work and get my life back to how it was.”

## 4. Discussion

The present study provides a multidimensional understanding of psychological flourishing among patients with burns. Participants described positive psychological experiences despite ongoing challenges such as pain, functional limitations, and changes in appearance, suggesting that flourishing does not necessarily imply the absence of distress. Rather, positive and negative experiences may coexist within the context of burn treatment and rehabilitation. Participants’ accounts reflected emotional, behavioral, interpersonal, meaning-related, and accomplishment-related dimensions of flourishing, illustrating that psychological well-being among this population extends beyond emotional recovery alone. These findings are broadly consistent with research on post-traumatic growth, which suggests that positive psychological experiences may coexist with distress following major traumatic events [43]. Overall, these findings suggest that, alongside attention to negative psychological symptoms, greater consideration could also be given to patients’ positive psychological experiences and resources during burn treatment and recovery.

Participants described positive emotional experiences in the context of treatment and rehabilitation, including renewed hope, optimism, and emotional stability. Some also reported reinterpreting their illness experiences through cognitive reappraisal and self-adjustment, with greater attention to wound healing and rehabilitation progress. These experiences may reflect a more positive cognitive orientation and can be interpreted through the Attention, Interpretation, and Memory (AIM) theory of well-being, which proposes that selective attention to and interpretation of positive information may contribute to subjective well-being [44,45]. From the perspective of the PERMA framework, these experiences illustrate how the positive emotion dimension may be expressed in the specific context of burn treatment and rehabilitation [26]. Previous studies involving organ transplant recipients and patients with chronic conditions have reported associations between higher levels of positive emotions and greater rehabilitation engagement and quality of life [46]. Similarly, Wu et al. (2026) found that positive psychological qualities played an important role in the illness-coping process of patients with moderate-to-severe burns, particularly through maintaining an optimistic attitude, finding hope in adversity, and enhancing resilience and adaptability [15]. Consistent with their findings, participants in the present study also described positive psychological experiences, including optimism and renewed hope. However, Wu et al. (2026) [15] primarily interpreted these positive psychological factors from the perspective of illness coping, regarding them as internal resources that helped patients manage disease-related threats. When viewed through the PERMA framework, our findings further suggested that positive emotional experiences extended beyond optimism and hope to include cognitive and emotional adjustment, acceptance of the burn experience, and greater emotional stability. These findings highlight the importance of addressing negative emotional states while also supporting positive emotional experiences in clinical nursing practice to facilitate patients’ psychological well-being.

Participants described active involvement in treatment and rehabilitation, including efforts to resume activities of daily living and maintain social interaction. These experiences reflected sustained engagement in recovery-related activities and were often accompanied by greater confidence, a stronger sense of control, and renewed participation in social roles. From the perspective of the PERMA framework, these experiences may represent context-specific manifestations of the engagement dimension [47]. They may also be interpreted in light of Bandura’s self-efficacy theory, which proposes that mastery experiences can strengthen individuals’ beliefs in their capabilities and support continued participation in goal-directed activities [48]. Previous studies have similarly suggested that sustained participation in physical activity and rehabilitation may be associated with improved emotional well-being and broader recovery outcomes [49,50]. Together, these findings suggest a possible interrelationship between perceived functional improvement and positive psychological experiences during burn recovery. Nevertheless, engagement in rehabilitation may be constrained by persistent pain, functional limitations, and fear of reinjury, which may contribute to fear-avoidance behaviors and reduced participation in rehabilitation and social activities [51,52]. These findings highlight the importance of addressing psychological as well as physical barriers throughout rehabilitation and supporting active participation through graded rehabilitation training and appropriate social support.

Participants described receiving multifaceted support from family members, healthcare professionals, peers, and broader social resources during treatment and rehabilitation. Within the PERMA framework, these experiences may be understood as context-specific manifestations of the relationships dimension, which emphasizes supportive interpersonal connections, belonging, and psychological security [26]. In the present study, family support emerged as an important source of both emotional and practical assistance. Participants described receiving daily care, emotional companionship, and financial support from family members, which contributed to a greater sense of security during rehabilitation. This finding may be related to the emphasis placed on family cohesion and family responsibility within the Chinese cultural context, in which family support has been closely associated with patients’ illness coping and rehabilitation experiences [53]. Support from healthcare professionals was also described as important. Professional guidance, encouragement, and positive communication were perceived as helping patients develop greater confidence in treatment and recovery. Deng et al. (2026) similarly reported that emotional and informational support from family members, friends, and social networks was associated with greater social participation among patients with burns [54]. Peer support also appeared to play an important role. Sharing experiences and emotions with other patients with burns contributed to feelings of being understood and accepted, which may help reduce loneliness and support coping and psychological adaptation [55]. These findings are consistent with the buffering model of social support, which proposes that social support may attenuate the adverse effects of stressors on psychological well-being and provide a protective resource for psychological adaptation [56]. Previous studies have also suggested that perceived social support may reduce stress responses by regulating neuroendocrine mechanisms and improving emotion regulation capacity [57]. The present study further showed that support extended beyond emotional and interpersonal resources. Economic and institutional support provided through social security systems was also perceived as important during rehabilitation. Because burn treatment and rehabilitation may impose substantial financial burdens, medical insurance, social assistance, and welfare policies may help reduce financial stress and provide greater stability during recovery [58]. Accordingly, clinical nursing practice should consider strengthening multilevel support by integrating family support, healthcare team support, and broader social resources. Such support may facilitate social adaptation and positive psychological development in burn patients.

Participants described changes in how they viewed life, health, family, and personal priorities following their burn experiences. These accounts included greater appreciation of health, increased attention to family, and a stronger sense of responsibility. Within the PERMA framework, such experiences can be understood as context-specific manifestations of the meaning dimension, which concerns a sense of purpose, value, and significance in life [47]. Some participants also described family responsibility as an important source of motivation for engaging in rehabilitation. Previous research has similarly suggested that major health events may prompt individuals to reconsider personal values and life goals and to report positive psychological changes [59]. Higher levels of meaning in life are also closely associated with improved subjective well-being, mental health, and psychological resilience [60,61]. Kim et al. (2025) reported that burn survivors reconstructed meaning in life through reflection on their traumatic experiences during rehabilitation [62]. Similarly, Chu et al. (2025) noted that although burn survivors face challenges such as changes in appearance, functional limitations, and social adaptation, they were still able to reaffirm their responsibilities and life values through positive reflection [63]. Related studies have further suggested that meaning in life is associated with motivation and emotion regulation and may support adaptive capacity in the face of adversity [64]. From a clinical perspective, nursing care should extend beyond physical recovery to support meaning-making and the re-establishment of family roles and responsibilities. Helping patients identify personally meaningful and achievable life goals may facilitate positive psychological functioning during recovery.

Participants described a sense of accomplishment associated with improvements in physical function, activities of daily living, and the resumption of social roles. Some also reported greater confidence and a stronger sense of control when achieving stage-specific rehabilitation goals and anticipating future recovery. Within the PERMA framework, these experiences can be understood as context-specific manifestations of the accomplishment dimension, which emphasizes goal attainment, competence, and personal growth [18]. Previous research has also shown that successful experiences can contribute to self-efficacy and strengthen individuals’ confidence in their abilities [65]. In addition, accomplishment experiences may improve subjective well-being by eliciting positive emotions and forming a positive developmental cycle [66]. Goal-setting theory further suggests that clear, achievable goals may strengthen behavioral motivation and support sustained engagement [67]. Participants described rehabilitation-related accomplishments ranging from completing basic daily activities to resuming social roles, which reflected perceived improvements in physical function. These experiences were also associated with greater hope and confidence regarding future life. The findings of the present study are consistent with those of Dukes et al. (2022) [14], who reported that a sense of accomplishment derived from completing daily activities or achieving rehabilitation milestones was associated with greater independence and perceived control. These experiences may, in turn, support more active engagement in rehabilitation. In practice, healthcare providers should consider setting individualized, stage-specific rehabilitation goals and providing timely positive feedback and encouragement to support patients’ sense of accomplishment. Such approaches may facilitate engagement in rehabilitation and psychological adaptation.

### 4.1. Recommendations for Further Research

Given regional variations in socioeconomic conditions, cultural contexts, and healthcare resources, future multicenter studies should recruit participants from diverse settings to examine the transferability of these findings. Longitudinal research is also needed to explore changes in psychological flourishing across different stages of burn recovery. Quantitative studies may further assess flourishing levels and associated factors, complementing the contextual insights provided by qualitative research. In addition, the PERMA framework may inform the development of structured positive psychological interventions, whose feasibility, acceptability, and effectiveness should be evaluated in future studies.

### 4.2. Strengths and Limitations

This study has several strengths. First, semi-structured in-depth interviews generated rich qualitative data on positive psychological experiences during burn rehabilitation, allowing for detailed exploration of patients’ subjective perceptions and meaning-making. Second, the PERMA framework served as a sensitizing theoretical lens that informed the interpretation of psychological flourishing experiences among patients with burns. The sample comprised participants with diverse ages, genders, and burn severity, which enhanced the heterogeneity and informational richness of the data. Moreover, by exploring an underexamined aspect of positive psychological experiences, this study provides additional empirical insights into psychological health among patients with burns.

This study has several limitations. First, participants were recruited from a single healthcare institution, which may limit the transferability of the findings to other cultural and healthcare settings. Second, the study used a non-longitudinal interview design and therefore could not capture potential changes in psychological flourishing experiences across different stages of treatment and recovery. Third, as a phenomenological qualitative study, this research primarily interpreted and elucidated experiences based on participants’ subjective accounts, and the findings do not involve causal inferences. Fourth, participant heterogeneity in demographic and clinical characteristics, including differences in the timing of interviews after burn injury, may have contributed to variation in flourishing experiences. Future studies with more homogeneous samples or specific clinical subgroups are warranted.

### 4.3. Relevance to Clinical Practice

The findings of this study suggest several considerations for clinical nursing practice. First, in addition to addressing psychological distress, healthcare professionals may also attend to patients’ positive psychological experiences and resources, including hope, engagement in rehabilitation, supportive relationships, meaning, and accomplishment. Second, the PERMA-related experiences identified in this study may provide a useful framework for recognizing patients’ positive psychological needs and informing individualized supportive care during burn recovery. Third, given the multidimensional support needs described by participants, healthcare professionals may consider strengthening coordination among family members, healthcare teams, peers, and broader social resources. Integrating these sources of support into routine care may help address patients’ psychological adaptation.

## 5. Conclusions

This phenomenological qualitative study identified multidimensional psychological flourishing experiences among patients with burns that can be interpreted through the PERMA framework. Positive psychological changes were identifiable among burn patients despite physical appearance alterations and functional limitations. The findings indicated that clinical nursing practice focused on negative psychological outcomes in burn patients can be complemented by interventions targeting the cultivation of positive psychological resources. The PERMA model can be applied to interpret the characteristics of psychological flourishing among burn patients and provide a theoretical framework for the development of positive psychological nursing interventions for this population.

## Figures and Tables

**Table 1:** Interview guide.

Outline	Core Question
Experience	Could you tell me about your experience of getting burned?
Theme: Flourishing	Our interview theme is “flourishing” mainly focusing on how this burn situation has affected your mental health status.
Relationships	After the burn injury, who has provided you with help or support? How have their attitudes influenced you?
Engagement	After the burn injury, what changes have occurred in your daily life and activities?
Positive Emotions	During your treatment or recovery process, how have you typically felt? What positive changes have you experienced?
Meaning	After experiencing the burn injury, what do you find most important in your life now? What is the most meaningful thing for you?
Achievement	During your recovery, have there been any things that made you feel a sense of achievement or satisfaction with yourself?

**Table 2:** General and disease-related data of burn patients.

ID	Sex	Age (Years)	Marital Status	Education	Occupation	Cause of Burn	TBSA (%)	Burn Site(s)	Burn Depth	Burn Severity	Burn Stage	Time Since Burn Injury
P1	Female	80	Married	JHS	Retired	Scald	7	2	1% Superficial II°; 5% Deep II°; 1% III°	Moderate	2	20 days
P2	Male	36	Married	JHS	Worker	Flame	30	2,3	30% III°	Extremely severe	2	37 days
P3	Male	49	Divorced	JHS	Self-employed	Flame	40	2,3,4	10% Superficial II°; 10% Deep II°; 20% III°	Extremely severe	3	49 days
P4	Male	28	Single	HS	Self-employed	Electrical injury	15	1,2	5% Deep II°; 10% III°	Severe	2	9 days
P5	Female	45	Married	CD	Employee	Scald	7	4	7% III°	Moderate	1	15 days
P6	Male	22	Single	CD	Food service worker	Flame	20	1,2,3,4	15% Superficial II°; 3% Deep II°; 2% III°	Severe	2	17 days
P7	Female	19	Single	CD	Student	Electrical injury	5	1	5% Deep II°	Moderate	3	31 days
P8	Male	60	Married	HS	Worker	Flame	10	1,2	8% Deep II°; 2% III°	Severe	2	18 days
P9	Male	54	Married	CD	Employee	Flame	3	2	1% Deep II°; 2% III°	Moderate	1	10 days
P10	Male	29	Single	JHS	Employee	Flame	19	1,2,3	19% III°	Extremely severe	3	>14 years
P11	Male	72	Married	PS	Retired	Flame	10	2,4	10% Deep II°	Moderate	2	25 days
P12	Female	36	Married	JHS	Employee	Scald	16	3,4	16% Superficial II°	Moderate	1	4 days
P13	Female	45	Married	PS	Worker	Flame	18	1,2,3	3% Superficial II°; 5% Deep II°; 10% III°	Severe	2	39 days
P14	Female	27	Single	JHS	Salesperson	Scald	5	1	5% III°	Moderate	3	>8 years
P15	Female	61	Married	JHS	Retired	Scald	8	1,2,4	6% Superficial II°; 1% Deep II°; 1% III°	Moderate	2	22 days
P16	Female	47	Married	CD	Unemployed	Flame	15	1,2,4,5	5% Deep II°; 10% III°	Severe	2	24 days
P17	Male	58	Married	JHS	Worker	Chemical exposure	95	1,2,3, 4,5,6	3% Deep II°; 92% III°	Extremely severe	3	139 days
P18	Female	22	Single	TSS	Employee	Flame	60	1,2,3, 4,5	10% Deep II°; 50% III°	Extremely severe	3	190 days
P19	Male	27	Single	BD	Employee	Electrical injury	1	2	1% III°	Moderate	1	8 days
P20	Male	60	Widowed	JHS	Cleaner	Chemical exposure	80	1,2,3,4	15% Deep II°; 65% III°	Extremely severe	3	62 days
P21	Male	41	Married	PS	Self-employed	Chemical exposure	15	1,2,3	14% Deep II°; 1% III°	Moderate	1	11 days
P22	Male	37	Divorced	JHS	Electrician	Electrical injury	8	1,2	7% Deep II°; 1% III°	Moderate	3	18 days

**Note:** TBSA = total body surface area. Education levels were coded as follows: PS = primary school; JHS = junior high school; HS = high school; TSS = technical secondary school; CD = college diploma; BD = bachelor’s degree. Burn sites were coded as follows: 1 = head, face, and neck; 2 = upper limbs; 3 = trunk; 4 = lower limbs; 5 = buttocks; 6 = perineum. Clinical burn stages were coded as follows: 1 = acute infection stage; 2 = wound healing stage; 3 = functional rehabilitation stage. Burn depth: Superficial II° = superficial partial-thickness burn; Deep II° = deep partial-thickness burn; III° = full-thickness burn. Time since burn injury refers to the elapsed time from the burn injury to the interview.

## Data Availability

The data generated and analyzed during this study are not publicly available due to privacy and ethical restrictions related to the qualitative interview data. Further information may be available from the corresponding author upon reasonable request, subject to ethical and confidentiality considerations.

## Supplementary Materials

The following supporting information can be downloaded at: https://www.mdpi.com/article/10.3390/healthcare14213633/s1. Supplementary Material File S1: COREQ Checklist.

## Abbreviations

The following abbreviations are used in this manuscript:

PERMA	Positive Emotion, Engagement, Relationships, Meaning, and Accomplishment
WHO	World Health Organization
COREQ	Consolidated Criteria for Reporting Qualitative Research
ADLs	Activities of Daily Living
