# Supplementary material for: Psychological Flourishing Experiences Among Burn Patients: A Phenomenological Qualitative Study Informed by the PERMA Framework
Source: Healthcare (Basel). 2026 Oct 27;14(21):3633. doi: 10.3390/healthcare14213633 (PMC13763451; doi:10.3390/healthcare14213633)
Supplement: Supplementary file 1 [file healthcare-14-03633-s001.zip › healthcare-4562571-supplementary.pdf]

Additional File S1: COREQ checklist.

No. Item	Guide questions/description	Reported
**Domain 1: Research team and reflexivity**		
Personal Characteristics		
1. Interviewer/facilitator	Which author/s conducted the interview or focus group?	DLZ, WLH, SYZ, HYL
2. Credentials	What were the researcher's credentials? E.g., PhD, MD	DLZ—Master<br>WLH—Master<br>SYZ—PhD<br>YW—Bachelor<br>HY—Master<br>LYC—Bachelor<br>MMP—Bachelor<br>HYL—Bachelor
3. Occupation	What was their occupation at the time of the study?	DLZ—Student<br>WLH—Student<br>SYZ—Chief Pharmacist<br>YW—Associate Chief Nurse<br>HY—Associate Chief Nurse<br>LYC—Chief Nurse<br>MMP—Associate Chief Nurse<br>HYL—Chief Nurse
4. Gender	Was the researcher male or female?	SYZ: Male<br>DLZ, WLH, YW, HY, LYC, MMP, HYL: Female
5. Experience and training	What experience or training did the researcher have?	DLZ—Burns department internship experience<br>WLH—Received qualitative research training and completed multiple qualitative studies<br>SYZ—Rich experience in burn medication management<br>YW—Rich experience in burn nursing<br>HY—Rich experience in burn nursing<br>LYC—Rich experience in burn nursing<br>MMP—Profound experience in wound care research<br>HYL—Extensive experience in

		nursing management and wound care research
Relationship with participants		
6. Relationship established	Was a relationship established prior to study commencement?	We did not establish a relationship with participants prior to study commencement.
7. Participant knowledge of the interviewer	What did the participants know about the researcher? E.g., personal goals, reasons for doing the research	Informed Consent Statement.
8. Interviewer characteristics	What characteristics were reported about the inter-viewer/facilitator? E.g., bias, assumptions, reasons and interests in the research topic	The interviewers' professional backgrounds and research experience relevant to burn care and qualitative research were reported in the manuscript and Title Page.
**Domain 2: study design**		
Theoretical framework		
9. Methodological orientation and Theory	What methodological orientation was stated to underpin the study? E.g., grounded theory, discourse analysis, ethnography, phenomenology, content analysis	Phenomenology.
Participant selection		
10. Sampling	How were participants selected? E.g., purposive, convenience, consecutive, snowball	Purposive.
11. Method of approach	How were participants approached? E.g., face-to-face, telephone, mail, email	Face-to-face.
12. Sample size	How many participants were in the study?	Twenty-two participants completed the study and were included in the final analysis.
13. Non-participation	How many people refused to participate or dropped out? Reasons?	Two of the 24 participants who initially agreed to participate withdrew during the interview process. One was unwilling to continue the in-depth discussion because of limited willingness to communicate, and the other experienced marked emotional distress when discussing personal experiences.

Setting		
14. Setting of data collection	Where was the data collected? E.g., home, clinic, workplace	Burn inpatient ward.
15. Presence of non-participants	Was anyone else present besides the participants and researchers?	No. All interviews were conducted one-to-one, with no family members or other non-participants present.
16. Description of sample	What are the important characteristics of the sample? E.g., demographic data, date	Results.
Data collection		
17. Interview guide	Were questions, prompts, and guides provided by the authors? Was it pilot tested?	Methods.<br>A pilot study was conducted with two interviewees.
18. Repeat interviews	Were repeat interviews carried out? If yes, how many?	Yes. One participant underwent a second interview to obtain richer data.
19. Audio/visual recording	Did the research use audio or visual recording to collect the data?	Audio Recording.
20. Field notes	Were field notes made during and/or after the interview or focus group?	Yes.
21. Duration	What was the duration of the interviews or focus group?	30 to 50 minutes.
22. Data saturation	Was data saturation discussed?	Yes.
23. Transcripts returned	Were transcripts returned to participants for comment and/or correction?	Yes.
**Domain 3: analysis and findings**		
Data analysis		
24. Number of data coders	How many data coders coded the data?	Two researchers independently coded the data, with disagreements resolved through discussion with a third researcher.
25. Description of the coding tree	Did authors provide a description of the coding tree?	Yes, in Figure 1.
26. Derivation of themes	Were themes identified in advance or derived from the data?	The themes were derived from the data with the guidance of the PERMA theoretical framework.
27. Software	What software, if applicable, was used to manage the data?	Excel.
28. Participant checking	Did participants provide feedback	Yes.

	on the findings?	
Reporting		
29. Quotations presented	Were participant quotations presented to illustrate the themes/findings? Was each quotation identified? E.g., participant number	Yes, themes are presented using quotes.<br>All quotations were identified by numbers.
30. Data and findings consistent	Was there consistency between the data presented and the findings?	Yes. In the result section, quotations are used to illustrate the consistency.
31. Clarity of major themes	Were major themes clearly presented in the findings?	Yes.
32. Clarity of minor themes	Is there a description of diverse cases or discussion of minor themes?	Yes, diverse cases were included, and minor themes were briefly discussed in the research.
